# ASSOCIATION OF RS2435357 AND RS1800858 POLYMORPHISMS IN RET PROTO-ONCOGENE WITH HIRSCHSPRUNG DISEASE: SYSTEMATIC REVIEW AND META-ANALYSIS

**DOI:** 10.1590/0102-672020190001e1448

**Published:** 2019-10-21

**Authors:** Abdolhamid AMOOEE, Mohamad Hosein LOOKZADEH, Seyed Reza MIRJALILI, Seyed Mohsen MIRESMAEILI, Kazem AGHILI, Masoud ZARE-SHEHNEH, Hossein NEAMATZADEH

**Affiliations:** 1 Shahid Sadoughi University of Medical Sciences, General Surgery;; 2 Shahid Sadoughi University of Medical Sciences, Pediatrics;; 3 Science and Art University, Biology;; 4 Shahid Sadoughi University of Medical Sciences, Radiology;; 5 Shahid Sadoughi University of Medical Sciences, Medical Genetics, Yazd, Yazd, Iran

**Keywords:** Hirschsprung disease, Polymorphism, Single Nucleotide, Meta-Analysis, Doença de Hirschsprung, Polimorfismo de nucleotídeo único, Metanálise

## Abstract

**Introduction:**

Many published studies have estimated the association of rs2435357 and rs1800858 polymorphisms in the proto-oncogene rearranged during transfection (RET) gene with Hirschsprung disease (HSCR) risk. However, the results remain inconsistent and controversial.

**Aim:**

To perform a meta-analysis get a more accurate estimation of the association of rs2435357 and rs1800858 polymorphisms in the RET proto-oncogene with HSCR risk.

**Methods:**

The eligible literatures were searched by PubMed, Google Scholar, EMBASE, and Chinese National Knowledge Infrastructure (CNKI) up to June 30, 2018. Summary odds ratios (ORs) and 95% confidence intervals (CIs) were used to evaluate the susceptibility to HSCR.

**Results:**

A total of 20 studies, including ten (1,136 cases 2,420 controls) for rs2435357 and ten (917 cases 1,159 controls) for rs1800858 were included. The overall results indicated that the rs2435357 (allele model: OR=0.230, 95% CI 0.178-0.298, p=0.001; homozygote model: OR=0.079, 95% CI 0.048-0.130, p=0.001; heterozygote model: OR=0.149, 95% CI 0.048-0.130, p=0.001; dominant model: OR=0.132, 95% CI 0.098-0.179, p=0.001; and recessive model: OR=0.239, 95% CI 0.161-0.353, p=0.001) and rs1800858 (allele model: OR=5.594, 95% CI 3.653-8.877, p=0.001; homozygote model: OR=8.453, 95% CI 3.783-18.890, p=0.001; dominant model: OR=3.469, 95% CI 1.881-6.396, p=0.001; and recessive model: OR=6.120, 95% CI 3.608-10.381, p=0.001) polymorphisms were associated with the increased risk of HSCR in overall.

**Conclusions:**

The results suggest that the rs2435357 and rs1800858 polymorphisms in the RET proto-oncogene might be associated with HSCR risk.

## RESUMO

**Introdução:**

Muitos estudos publicados estimaram a associação dos polimorfismos rs2435357 e rs1800858 do proto-oncogene rearranjado durante a transfecção (RET) com o risco de doença por Hirschsprung (HSCR). No entanto, os resultados permanecem inconsistentes e controversos.

**Objetivo:**

Realizar metanálise para obter estimativa mais precisa da associação dos polimorfismos rs2435357 e rs1800858 no proto-oncogene RET com risco de HSCR.

**Método:**

A literatura elegível foi pesquisada pelo PubMed, Google Scholar, EMBASE e CNKI até 30 de junho de 2018.

**Resultados:**

Um total de 20 estudos, incluindo dez (1.136 casos 2.420 controles) para rs2435357 e dez (917 casos 1.159 controles) para rs1800858 foram incluídos. Os resultados globais indicaram que o rs2435357 (modelo alelo: OR=0,230, IC 95% 0,178-0,298, p=0,001; modelo homozigoto: OR=0,079, IC 95% 0,048-0,130, p=0,001; modelo heterozigoto: OR=0,149 , IC 95% 0,048-0,130, p=0,001, modelo dominante: OR=0,132, IC 95% 0,098-0,179, p=0,001 e modelo recessivo: OR=0,239, IC 95% 0,161-0,353, p=0,001) e rs1800858 (modelo alelo: OR=5,594, IC 95% 3,653-8,877, p=0,001; modelo homozigoto: OR=8,453, IC 95% 3,783-18,890, p=0,001; modelo dominante: OR=3,469, IC 95% 1,881- 6,396, p=0,001 e modelo recessivo: OR=6,120, 95% CI 3,608-10,381, p=0,001) polimorfismos foram associados com o aumento do risco de HSCR em geral.

**Conclusões:**

Os resultados sugerem que os polimorfismos rs2435357 e rs1800858 no proto-oncogene RET podem estar associados ao HSCR**.**

## INTRODUCTION

Hirschsprung disease (HSCR), also known as congenital megacolon, is a life-threatening birth defect characterized by the absence of enteric ganglia in the submucosal and myenteric plexuses of the gastrointestinal tract^6^. It´s incidence varies from 1:5,000 to 1:10,000 live births with an overall male to female ratio of 3:1 to 5:1, particularly in those with short segments^16^ ^,^ ^21^. The diagnosis is established in 15% within the first month of life, in 40-50% in the first three months, in 60% at the end of the first year of age, and in 85% by four years^7^. The exact mechanism of HSCR is unknown, but it is clear that both genetic and environmental factors are involved^6^. Trisomy chromosome 21 is the most frequent chromosomal abnormality (>90% cases) associated with HSCR disease^1^. Furthermore, it is associated with other congenital malformations in 5%-32% of cases including gastrointestinal tract, by CNS anomalies, hearing impairment and congenital anomalies of the kidney and urinary tract^28^ ^,^ ^31^. Perinatal and environmental risk factors for HSCR, such as vitamin A, maternal age, obesity, parity, hypothyroidism during pregnancy, medical drug use have been sparsely studied; however, the results have not been consistent^15^ ^,^ ^22^ ^,^ ^43^.

HSCR can be inherited as an autosomal dominant, autosomal recessive and even as a polygenic disorder^1^. However, approximately in 30% of cases, it is associated with other malformations^8^. Genetic association analyses have identified 12 susceptibility loci including EDNRB, EDN3, GDNF, NTN, SOX10, PHOX2B, ECE1, KIAA1279/KBP, ZFHX1B, TTF-1 and NRG1^13^. However, variations in most of these loci are found mostly in the syndromic cases, in which HSCR is associated with other congenital malformation^1^ ^,^ ^8^. Linkage analyses of multiplex HSCR families established that the proto-oncogene rearranged during transfection (RET) is the major susceptibility gene to its development^8^. RET is a trans-membrane tyrosine kinase receptor which also involved in multiple endocrine neoplasia type 2 (MEN 2), causing medullary thyroid carcinoma, pheochromocytoma and primary hyperparathyroidism^18^ ^,^ ^25^. Among the variations of RET gene, rs2435357 and rs1800858 polymorphisms, locating in intron 1 and exon 2 of the RET gene, respectively, have been wildly investigated in HSCR. However, the results from different studies are controversial^8^. 

Therefore, we carried out current systemic review and meta-analysis to clarify the associations of the SNP rs2435357 and rs1800858 with susceptibility to HSCR.

## METHOD

### Literature search strategy

A comprehensive literature search was performed using PubMed, EMBASE, Google scholar, Chinese National Knowledge Infrastructure (CNKI), Chinese Biomedical, WanFang and VIP database to identify all eligible studies evaluating the association of the RET rs2435357 and rs1800858 polymorphisms with HSCR risk up to June 30, 2018. The key words were as follows: (‘’Hirschsprung disease’’ OR ‘’HSCR’’ OR ‘’congenital megacolon’’) AND (‘’Rearrange during Transfection’’ OR ‘’RET Proto-oncogene’’ OR ‘’Proto-Oncogene C-Ret’’ OR ‘’RET gene’’ OR ‘’Cadherin-Related Family Member 16’’ OR ‘’Cadherin Family Member 12’’) AND (‘’rs1800858’’ OR ‘’c.135G>A’’ OR ‘’Ala45Ala’’) AND (‘’rs2435357’’ OR ‘’IVS1+9277C>T’’ OR ‘’c.73+9277C>T’’) AND (“polymorphism” OR “SNPs” OR “variation” OR “locus” OR “mutation”). The search was limited to human studies and published studies. In addition, the references list of relevant case-control studies and reviews were manually searched to identify any additional eligible studies. If two or more studies had the same or overlapping data, only the study with the largest sample or most recently published study was included in the meta-analysis.

### Data collection

The data from the relevant published studies were extracted independently by two of the authors and entered them in a customized questionnaire. Then, the extracted data were compared, and disagreements were resolved through a discussion between the two researchers. For each eligible study, the following data were extracted: first author’s name, publication year, country of origin, ethnicity, genotyping methods, source of controls (population-based and hospital-based), case and control numbers, genotype frequency of SNPs, minor allele frequency in controls, and Hardy-Weinberg equilibrium (HWE) in controls. The ethnicity was divided into Asian and Caucasian or others. In addition, studies was performed on different populations were considered as independent studies.

### Inclusion and exclusion criteria

Selected studies were included in the meta-analysis if they met the following criteria: 1) case-control or cohort studies; 2) evaluating the association of the rs2435357 and rs1800858 polymorphisms of RET gene with susceptibility to HSCR; 3) studies with sufficient data to perform a meta-analysis. Accordingly, studies with the following characteristics were excluded: 1) not case-control or cohort study; 2) no control population; 3) studies with insufficient available data or lacking of genotypes distribution data; 4) abstracts, comments, case reports, letters, editorials, reviews, and systematic reviews; 5) published studies containing duplicate data.

### Statistical analysis

The strength of association of RET rs2435357 and rs1800858 polymorphisms with HSCR risk was measured by odds ratios (ORs) with 95% confidence intervals (CIs). Statistical significance of the summary OR was determined using the Z-test. We used five models to evaluate associations of the RET rs2435357 and rs1800858 polymorphisms with HSCR risk including: allele model (B vs. A), homozygous model (BB vs. AA), heterozygous model (BB vs. BA), dominant model (BB+BA vs. AA), and recessive model (BB vs. AA+BA). The heterogeneity between studies was evaluated by chi-squared based Q test, in which a p-value less than <0.05 was considered obvious heterogeneity. In addition, the I2 value was used to test the degree of heterogeneity, in which I2<25%, no heterogeneity; I2 25-50%, moderate heterogeneity; I2>50%, large or extreme heterogeneity^17^. The fixed effects model was used to pool ORs and 95% confidential interval (CI) when there was no significant heterogeneity. Otherwise, the random effects model (the DerSimonian and Laird method) was used. Hardy-Weinberg equilibrium was assessed by the goodness-of-fit Chi-square test. A sensitivity analysis was mainly performed by omission of a single study each time to assess the stability of obtained pooled ORs. In addition, sensitivity analyses were performed by omission HWE-violating studies. The potential publication bias was estimated by the funnel plot, in which the standard error of log (OR) of each study was plotted against its log (OR). In addition, Funnel plot asymmetry was further assessed by the method of Egger’s linear regression test, in which p<0.05 was considered a significant publication bias. The quality of genotype data was estimated by Hardy-Weinberg equilibrium (HWE) and low quality studies deviated from HWE were excluded in the sensitivity analysis. All the tests in this meta-analysis were conducted with Comprehensive meta-analysis CMA software (version 2.0; College Station, TX). P-values<0.05 were considered statistically significant. Ethical approval was not necessary, as this was a meta-analysis based on previous studies, and no direct handing of personal data or recruitment of participants.

## RESULTS

### Study characteristics

**FIGURE 1:**
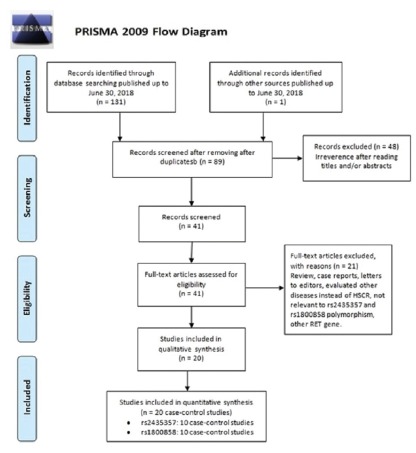
Flow chart depicting exclusion/inclusion of individual studies for meta-analysis

Following the online search of multiple databases, 131 potentially relevant publications were retrieved. As shown in Figure 1, after excluding the duplicates, 89 publications were remained. Among them, 69 publications were excluded because they were irrelevant, reviews/abstracts, not about human subjects, or not published in English. Finally, 20 case-control studies, including nine with 1,136 HSCR cases 2,420 controls for rs2435357 ^3^ ^,^ ^14^ ^,^ ^19^ ^,^ ^27^ ^,^ ^30^ ^,^ ^32^ ^,^ ^40^ ^,^ ^41^ ^,^ ^42^, and ten with 917 HSCR cases 1,159 controls for rs1800858 ^5^ ^,^ ^9^ ^,^ ^10^ ^,^ ^12^ ^,^ ^23^ ^,^ ^24^ ^,^ ^30^ ^,^ ^34^ ^,^ ^37^ ^,^ ^39^ were included. The characteristics of each study are summarized in Table 1. Among 18 case-control studies, 14 were conducted in Asians and four in Caucasians. All the included studies were published between 2003 and 2017. The HSCR cases sample size ranged from 16 to 362. Genotyping methods used included PCR, PCR-RFLP, TaqMan assay, and PCR-HRM. Fourteen matching for the controls were population-based, two were hospital-based, and two did not stated. All studies showed that the distribution of genotypes in the control group was in agreement with the HWE (p<0.05), except for two studies^22^ ^,^ ^23^ for rs2435357 and two^28^ ^,^ ^29^ for rs1800858 polymorphisms.

**TABLE 1:** Main characteristics of studies included in this meta-analysis

First Author/Year	Country (Ethnicity)	Genotyping Technique	SOC	Case/Control	Cases			Controls					MAFs		HWE	
					Genotypes			Allele		Genotypes			Allele			
rs2435357					TT	TC	CC	T	C	TT	TC	CC	T	C		
Zhang 2007	China (Asian)	PCR	HB	99/132	57	28	14	142	56	29	62	41	120	144	0.545	0.544
Arnold 2008	European*	TaqMan	HB	62/30	12	27	23	51	70	2	14	14	18	42	0.700	0.542
Miao 2010	China (Asian)	PCR	HB	315/352	228	65	22	521	109	62	169	95	293	359	0.550	0.390
Phusantisampan 2012	Thailand (Asian)	PCR-RFLP	HB	68/120	47	14	7	108	28	31	64	25	126	114	0.475	0.447
Prato 2009	Italy (Caucasian)	PCR	HB	22/85	11	6	5	28	16	3	32	50	38	132	0.776	0.435
Zhang 2015	China (Asian)	TaqMan	NS	59/59	42	16	1	100	18	13	30	16	56	62	0.525	0.880
				76/59	59	15	2	133	19							
Gunadi 2016	Indonesia (Asian)	PCR-RFLP	NS	93/136	67	22	4	156	30	27	83	26	137	135	0.496	0.010
Yang 2017	China (Asian)	TaqMan	PB	362/1448	209	126	27	544	180	329	802	317	1460	1436	0.495	=0.001
Li 2017	China (Asian)	TaqMan	NS	99/114	69	27	3	165	33	19	58	37	96	132	0.578	0.641
rs1800858					GG	AG	AA	G	A	GG	AG	AA	G	A		
Fitze 2003	German (Caucasian)	NS	HB	80/120	10	30	40	50	110	65	47	8	177	63	0.262	0.899
Garcia-Barcelo 2005	China (Asian)	PCR-RFLP	HB	172/194	14	40	118	68	276	58	100	36	216	172	0.443	0.536
Burzynski 2004	Netherland (Caucasian)	NS	HB	105/126	21	27	57	69	141	77	40	9	184	58	0.230	0.242
Zhang 2005	China (Asian)	PCR	HB	16/40	2	1	13	5	27	15	12	13	42	38	0.475	0.011
Du 2006	China (Asian)	PCR	HB	94/122	4	33	57	41	147	13	88	21	144	130	0.532	=0.001
Liu 2008	China (Asian)	LDR	PB	116/144	11	42	63	64	168	42	73	29	157	131	0.454	0.789
Saryono 2010	Indonesia (Asian)	PCR-RFLP	PB	54/46	5	23	26	33	75	10	30	6	5	23	0.456	0.033
Liu 2010	China (Asian)	PCR	HB	125/148	12	45	68	69	181	43	75	30	161	135	0.456	0.794
Tou 2011	China (Asian)	PCR	HB	123/168	10	32	81	52	194	52	85	31	10	32	0.437	0.716
Phusantisampan 2012	Thailand (Asian)	PCR-RFLP	HB	68/120	36	23	9	95	41	40	51	29	36	23	0.454	0.117

* Authors declared that the ancestry of the participants was European (Caucasians); PCR=polymerase chain reaction restriction; PCR-RFLP=polymerase chain reaction restriction fragment length polymorphism; LDR=ligase detection reaction; HB=hospital based; PB= population based; NS=not stated; MAFs=minor allele frequencies; HWE=hardy-weinberg equilibrium.

### Quantitative data synthesis

#### rs2435357

**FIGURE 2:**
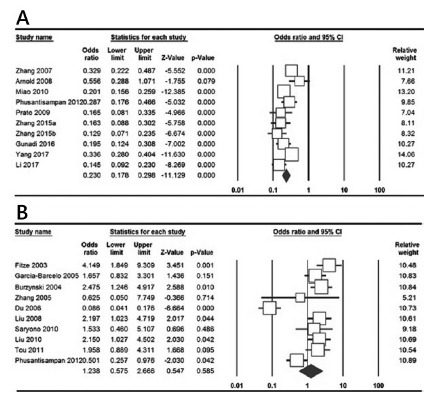
Forest plots of rs2435357 and rs1800858 polymorphisms in the RET gene and HSCR risk: A) rs2435357 (allele model: C vs. T); B) rs1800858 (heterozygote model: AG vs. GG)

Table 2 listed the main results of the meta-analysis of rs2435357 polymorphism in the RET proto-oncogene and HSCR risk. We pooled all the ten case-control studies to assess the overall association of rs2435357 polymorphism with HSCR risk. Overall pooled analysis suggest a significant association between rs2435357 polymorphism and HSCR risk in overall estimations under all five genetic models, i.e., allele (C vs. T: OR=0.230, 95% CI 0.178-0.298, p=0.001, Figure 2A), homozygote (CC vs. TT: OR=0.079, 95% CI 0.048-0.130, p=0.001); heterozygote (CT vs. TT: OR=0.149, 95% CI 0.048-0.130, p=0.001); dominant (CC+CT vs. TT: OR=0.132, 95% CI 0.098-0.179, p=0.001); and recessive (CC vs. CT+TT: OR=0.239, 95% CI 0.161-0.353, p=0.001).

**TABLE 2:** Results of the association of RET polymorphism with OA risk

Subgroup	Genetic Model	Type of Model	Heterogeneity		Odds Ratio				Publication Bias	
			I2 (%)	PH	OR	95% CI	Ztest	POR	PBeggs	PEggers
rs2435357										
Overall	C vs. T	Random	74.18	=0.001	0.230	0.178-0.298	-11.129	=0.001	0.858	0.209
	CC vs. TT	Random	60.85	0.006	0.079	0.048-0.130	-10.008	=0.001	0.371	0.178
	CT vs. TT	Random	58.02	0.011	0.149	0.108-0.205	-11.670	=0.001	1.000	0.156
	CC+CT vs. TT	Random	59.29	0.009	0.132	0.098-0.179	-13.220	=0.001	0.371	0.068
	CC vs. CT+TT	Random	52.17	0.027	0.239	0.161-0.353	-7.184	=0.001	0.107	0.219
rs1800858										
Overall	A vs. G	Random	89.58	=0.001	5.594	3.653-8.877	7.679	=0.001	0.210	0.469
	AA vs. GG	Random	86.57	=0.001	8.453	3.783-18.890	5.203	=0.001	0.591	0.934
	AG vs. GG	Random	88.56	=0.001	1.238	0.575-2.666	0.547	0.585	1.000	0.883
	AA+AG vs. GG	Random	83.71	=0.001	3.469	1.881-6.396	3.984	=0.001	0.591	0.800
	AA vs. AG+GG	Random	83.23	=0.001	6.120	3.608-10.381	6.720	=0.001	1.000	0.798

#### rs1800858

Table 2 listed the main results of the meta-analysis of rs1800858 polymorphism in the RET proto-oncogene and HSCR risk. Overall pooled analysis suggest a significant association of rs1800858 polymorphism and HSCR risk under four genetic models, i.e., allele (A vs. G: OR=5.594, 95% CI 3.653-8.877, p=0.001); homozygote (AA vs. GG: OR=8.453, 95% CI 3.783-18.890, p=0.001); dominant (AA+AG vs. GG: OR=3.469, 95% CI 1.881-6.396, p=0.001); and recessive (AA vs. AG+GG: OR=6.120, 95% CI 3.608-10.381, p=0.001), but not under heterozygote model (AG vs. GG: OR=1.238, 95% CI 0.575-2.666, p=0.585, Figure 2B).

### Sensitivity analysis

The sensitivity analysis was conducted by omitting each study in each genetic model or removing certain studies such as those studies that did not conform to HWE. After individual study omission, the corresponding pooled OR was not altered significantly. This indicates that our results are statistically robust under all five genetic models examining associations of rs2435357 and rs1800858 polymorphisms with HSCR risk.

### Publication bias

**FIGURE 3:**
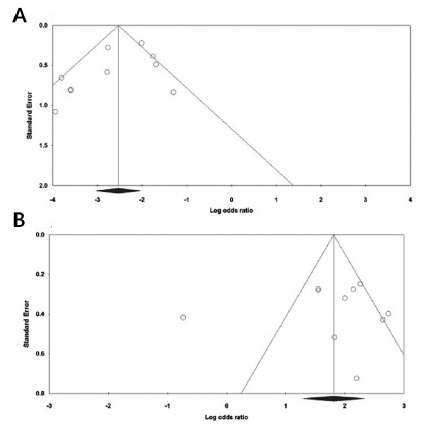
Funnel plot for the detection of the publication bias for association of rs2435357 and rs1800858 polymorphisms in the RET gene with HSCR risk;a random-effects model was used: A) rs2435357 (homozygote model: CC vs. TT); B) rs1800858 (recessive model: AA vs. AG+GG).

Begg’s funnel plot and Egger’s test were performed to assess the possible publication bias of included studies. The shapes of the Begg’s funnel plot did not reveal any evidence of obvious asymmetry under all five genetic models. In addition, Egger’s linear regression also did not show any significantly statistical evidence of publication bias for rs2435357 under all five genetic models, i.e., allele (C vs. T: P_Beggs_=0.858 and P_Eggers_=0.209), homozygote (CC vs. TT: P_Beggs_=0.371 and P_Eggers_=0.178, Figure 3A), heterozygote (CT vs. TT: P_Beggs_=1.000 and P_Eggers_=0.156), dominant (CC+CT vs. TT: P_Beggs_=0.371 and P_Eggers_=0.068) and recessive (CC vs. CT+TT: P_Beggs_=0.107 and P_Eggers_=0.219). Moreover, the Egger’s test did not reveal publication bias rs1800858 polymorphism under all five genetic models, i.e., allele (A vs. G: P_Beggs_=0.210 and PEggers=0.469), homozygote (AA vs. GG: P_Beggs_=0.591 and P_Eggers_=0.934), heterozygote (AG vs. GG: P_Beggs_=1.000 and P_Eggers_=0.883), dominant (AA+AG vs. GG: P_Beggs_=0.591 and P_Eggers_=0.800) and recessive (AA vs. AG+GG: P_Beggs_=1.000 and P_Eggers_=0.798, Figure 3B).

## DISCUSSION

The gene for RET proto-oncogene, members of the glial cell line-derived neurotrophic factor (GDNF) family, maps to chromosome 10q^11.21^, contains 21 exons and covers 60kbp DNA^36^. The RET proto-oncogene encode a trans-membrane receptor tyrosine kinase protein with an extracellular domain rich in cysteine and an intracellular domain enriched in tyrosine that is important in transferring cell growth and differentiation signals^33^. The RET proto-oncogene germline loss of function mutations are associated with the development of HSCR, while gain of function mutations are responsible for development of various types of human cancer, including medullary thyroid carcinoma, multiple endocrine neoplasia type 2 (MEN 2) and 2B, pheochromocytoma and parathyroid hyperplasia^4^. To date, several genotype-phenotype correlations have been defined in association of RET mutations with different variants of MEN2 syndrome including MEN 2A, MEN 2B, and familial medullary thyroid carcinoma (FMTC)^38^.

Several studies have been published exploring the association of the rs2435357 and rs1800858 polymorphisms in RET proto-oncogene with HSCR risk. However, the results of those studies were inconsistent and inconclusive, due to the ethnic differences and small sample size. Therefore, meta-analysis as a powerful tool for summarizing the different studies results is needed to achieve a more comprehensive and reliable conclusion on both polymorphisms in order to provide further insights into this debated subject. This meta-analysis and systematic review, including ten studies with 1,136 cases 2,420 controls for rs2435357 and ten with 917 cases 1,159 controls for rs1800858 were identified and analyzed in this meta-analysis. We found that of rs2435357 and rs1800858 polymorphisms in RET gene are associated with the HSCR risk. These findings are consistent with the meta-analysis by Liang et al^20^. They performed a meta-analysis on association of rs2435357 polymorphism with five studies (566 cases and 719 controls) and rs1800858 polymorphism with nine studies (863 cases and 1,118controls) with HSCR risk. They found rs2435357 and rs1800858 polymorphisms of RET are associated with susceptibility to HSCR. However, their meta-analysis the sample size is rather small and not adequate enough to detect the possible associations.

Between-study heterogeneity is common in meta-analyses, and identifying potential sources of heterogeneity is an essential component of a meta-analysis^26^ ^,^ ^35^. The most potential sources of heterogeneity in a genetic association meta-analysis are study design, ethnicity, genotyping methods, source of controls, and so on^2^ ^,^ ^11^ ^,^ ^29^. Selection bias, although no publication bias was observed, is a possible major source of heterogeneity. Therefore, we have performed subgroup analysis and sensitivity analysis by removing HWE-violating studies to found out source of heterogeneity in this meta-analysis. However, heterogeneity before and after the subgroup analysis and process of individual study removing did not reduced or disappeared. Thus, this finding confirmed that the meta-analysis results were statistically robust and that our results were reliable and stable.

This study has two main advantages were as: first, this was the most accurate and comprehensive meta-analysis on rs2435357 and rs1800858 polymorphisms of RET with HSCR risk; second, no publication bias was observed in the present meta-analysis results indicating that our results might be unbiased. However, there were some limitations to this study that may have affected our conclusions. First, the present meta-analysis was limited by relatively small number of studies and sample size on both rs2435357 and rs1800858 polymorphisms, which thus leading to smaller studies in subgroup analysis and weaken statistical power; thus, needs further studies. Second, only studies on Asians and Caucasians populations were involved in this meta-analysis. This bias may exist because we could not determine the role of rs2435357 and rs1800858 polymorphisms in whole populations. Thus, studies on other ethnicities such as Africans and Latinos must be performed to determine the potential effects of ethnic variation on HSCR susceptibility. Third, we have included only the data of published studies, publication bias may be exist, although our results of publication bias tests showed no significance. Fourth, because relevant information was insufficient in the original data, we did not perform stratification analysis by other covariates such as age, gender and so on. This might has caused confounding bias. Finally, it is known that the HSCR has a multifactorial etiology of involving in gene-gene, and gene environment interactions. However, these interactions could not be investigated in the present meta-analysis due to no appropriate data.

## CONCLUSION

This meta-analysis suggested that the rs2435357 and rs1800858 polymorphisms in RET proto-oncogene may be associated with susceptibility to HSCR. However, because of the relatively small size of included studies, future large-scale studies on different ethnicity are needed to confirm these findings.
